# Research on Improved DNA Coding and Multidirectional Diffusion Image Encryption Algorithm

**DOI:** 10.3390/e25050746

**Published:** 2023-05-01

**Authors:** Jia Liu, Haiping Chang, Weiyu Ran, Erfu Wang

**Affiliations:** Electrical Engineering College, Heilongjiang University, Harbin 150080, China; 2201653@s.hlju.edu.cn (J.L.); 2201651@s.hlju.edu.cn (H.C.); 2201709@s.hlju.edu.cn (W.R.)

**Keywords:** image encryption, DNA coding, multidirectional diffusion

## Abstract

In order to make the security and operating efficiency of an image encryption algorithm coexist, this study proposed a color image encryption algorithm with improved DNA coding and rapid diffusion. During the stage of improving DNA coding, the chaotic sequence was used to form a look-up table to complete the base substitutions. In the replacement process, several encoding methods were combined and interspersed to make the randomness higher, thereby improving the security performance of the algorithm. In the diffusion stage, three-dimensional and six-directional diffusion was performed on the three channels of the color image by taking the matrix and the vector as the diffusion unit successively. This method not only ensures the security performance of the algorithm, but also improves the operating efficiency in the diffusion stage. From the simulation experiments and performance analysis, it was shown that the algorithm has good encryption and decryption effects, large key space, high key sensitivity, and strong security. The algorithm can effectively resist differential attacks and statistical attacks, and has good robustness.

## 1. Introduction

People are constantly exchanging information using the Internet and smartphones due to the rapid expansion of the Internet and the popularity of smartphones. While there is a lot of information sharing and convenience, a lot of data have been leaked, tampered with, and counterfeited. As an essential carrier of information interaction, digital images mainly comprise grayscale and color images. Compared with grayscale images, color images contain more information and account for most digital images. As a result, developing a good color image encryption technique is critical.

Encryption technology transforms ordinary images into noise-like or texture-like secret images, using keys and encryption algorithms to provide safe communication. The original image can only be recovered after decryption if the proper key is obtained. At the same time, because of the effect and interference of equipment, lighting, and other settings, encrypted images are easily lost or contaminated by numerous sounds when transferred via networks, which directly impacts whether the encrypted images can be correctly decoded. Encryption security is lost when data are intercepted, manipulated, or deciphered.

It is a matter of time before any intercepted image data are deciphered. In order to ensure that the encrypted images are adequately safe, information embedding can be chosen to decrease third-party attention. Of course, the most straightforward approach is to make the encryption algorithm sophisticated enough, the key space wide enough, and the method robust enough to withstand brute force attacks, differential attacks, statistical attacks, and others. Therefore, an encryption algorithm that is complex enough to ensure operational efficiency and has strong anti-attack and anti-interference performance has always been the research goal.

In recent years, developing a means to ensure the secure storage and transmission of digital photographs over the Internet has become a research hotspot [1,2,3]. Image data have characteristics of a two-dimensional structure, high redundancy, and large data volume. Thus, encryption algorithms such as 3DES and AES are no longer applicable. Image encryption algorithms mainly include scrambling, replacement, and diffusion algorithms. Among the scrambling methods, encryption algorithms such as Zigzag scan [4,5,6], Arnold transform [7,8], and magic square transform [9] can successfully disrupt visual information; however, they have difficulty withstanding modern cryptanalysis tools. DNA is a natural information storage medium with the advantages of high storage density, long storage time, and low loss rate [10,11]. Researchers have found that data can be converted into sequence information of different bases in the DNA molecular chain through DNA algorithms for replacement and storage, especially in image processing [12,13,14]. However, the traditional DNA encoding algorithm is weak in its anti-exhaustive attack ability, due to its fixed DNA base complementary pairing criterion and base operation criterion, and is prone to security risks. Diffusion technology hides the information of plaintext pixels in as many pixels as possible, without changing the position of the pixels, to improve the security of the encryption scheme. However, the traditional one-dimensional diffusion encryption algorithm is inefficient when dealing with images that have large amounts of data. 

It has been discovered that chaotic systems that are highly sensitive to changes in initial values produce a significant number of excellent pseudorandom sequences that naturally comply with the laws of scrambling, substitution, and diffusion [15,16,17,18,19,20,21]. As chaotic anti-control technology matures, more researchers are committed to harnessing chaotic systems to build novel encryption algorithms that totally conceal the statistical properties of original and encrypted images. Pak [22] created a simple and effective chaotic system using the output sequence difference of two identical one-dimensional chaos maps, which can provide a one-dimensional chaotic system with superior chaotic performance and a longer chaotic range than prior chaotic maps. Ratna [23] proposed an image encryption algorithm based on a chaotic system and DNA sequence operation that uses wave transmission properties to develop a new DNA horizontal wave displacement scheme and horizontal progressive image diffusion method that can effectively resist chosen plaintext attacks and known plaintext attacks. Liu [24] encoded, obfuscated, and diffused digital images via hyperchaotic dynamic DNA mechanisms. This encryption procedure is robust, since dynamic DNA diffusion is estimated in the blocks.

The hyperchaotic system has a complex structure, can stretch and fold in numerous directions, and can keep good random features in the digital system. Nevertheless, the operational efficiency is considerably lowered while improving safety performance, and the practical value is low. Jin Jianguo [25] increased the algorithm’s security using chaotic dynamic randomization and random modulation FRFT rotation factor; however, the time complexity is significant. Although Zhao’s [26] ciphertext block encryption mode maintains the algorithm’s security performance, the proposed diffusion mechanism is complex, resulting in low operating efficiency. Ünal [27] partitioned multi-threaded pictures, which considerably improves algorithm efficiency but does not provide a spreading method between separate threads, making differential attacks harder to resist. Due to the disadvantages of existing picture encryption methods, such as slow and insufficient pixel diffusion speed, Ge Bin [28] presented a four-way diffusion approach to boost the diffusion speed of the matrix rapidly.

In order to make the encryption algorithm have sufficient security and operating efficiency, based on the four-dimensional hyperchaotic system, this study proposed an improved DNA encoding algorithm and a three-dimensional six-way diffusion algorithm. In DNA encoding, the chaotic sequence is used to generate a lookup table, and the base substitution is realized by indexing the base pairs stored in the lookup table. In the diffusion stage, the three channels of the color image are rapidly diffused with a matrix and vector as the diffusion unit. This method ensures the complexity of the algorithm, and improves its operating efficiency in the diffusion stage.

## 2. Previous Theoretical Analyses

### 2.1. Arnold Mapping

Vladimir Igorevich Arnold, a Russian mathematician, proposed Arnold mapping. Since Arnold frequently used cat photos as examples when teaching, the technique is known as “cat mapping.” This mapping approach scrambles the position of each pixel in an image by continuously folding, stretching, and transforming in a small area.

**Figure 1:**
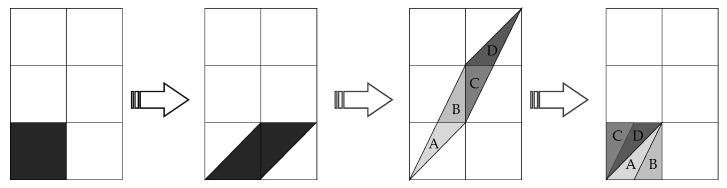
Changes in Arnold map.

As a mainstream dislocation approach, the algorithm is defined as follows, and its mapping variation is shown in Figure 1.

The pixel blocks in Figure 1 are stretched and distributed to other areas, such as A, B, C, D, and then folded back to the original pixel blocks.

(1) $$\left[\begin{array}{c} x_{n+1}\\ y_{n+1} \end{array}\right]=\left[\begin{array}{cc} 1 & a\\ b & ab+1 \end{array}\right]\left[\begin{array}{c} x_{n}\\ y_{n} \end{array}\right]\mathrm{mod}\left(N\right),$$

where *a*, *b*, and N are positive integers, and N is the width of the square matrix, *x_n_* and *y_n_* represent the position of pixels in the grayscale image before transformation, while *x_n_*_+1_ and *y_n_*_+1_ represent the positions of pixels after transformation.

Figure 1 shows that the pixel position of the input image is extended by multiplying the matrix, and then the position is modulo the square matrix width of the stretched value returned to the original matrix. At the same time, Arnold mapping is a one-to-one mapping, meaning that each point in the matrix will shift to another point in the matrix to fulfill the goal of pixel position diffusion.

To restore the scrambled position, Arnold mapping inverse change can be performed using Equation (2):

(2) $$\left[\begin{array}{c} x_{n+1}\\ y_{n+1} \end{array}\right]=\left[\begin{array}{cc} ab+1 & -b\\ -a & 1 \end{array}\right]\left[\begin{array}{c} x_{n}\\ y_{n} \end{array}\right]\mathrm{mod}\left(N\right),$$

where parameters *a*, *b* and N are consistent with Formula 1, *x_n_* and *y_n_* represent the position of a pixel in the grayscale image after replacement, and *x_n_*_+1_ and *y_n_*_+1_ represent the position of a pixel restored.

Although the Arnold mapping algorithm is basic and quick to implement, it is confined to square matrix transformations and has the flaws of periodicity and a fixed starting point.

### 2.2. Hyperchaotic Lorenz System

The hyperchaotic Lorenz system is an evolution of the 3DLorenz system. Linear feedback is added on the basis of the 3DLorenz system, as shown in Equation (3):

(3) $$\{\begin{array}{c} \frac{dx}{dt}=a\left(y-x\right)+w\\ \frac{dy}{dt}=cx-y-xz\\ \frac{dz}{dt}=xy-bz\\ \frac{dw}{dt}=-yz+rw \end{array}$$

where, *a*, *b*, *c*, and *r* are the coefficients of the 4DLorenz system. When a=10, b=8/3, c=28, −1.52≤r, or r≥−0.06, the system is in a hyperchaotic state. When r=−1, there are four Lyapunov exponents in this system, which are 0.3381, 0.1586, 0, and −15.1752, with two of them being bigger than zero. The high-dimensional hyperchaotic system has a more complicated structure than the low-dimensional chaotic system, and the generated sequence is more unpredictable, making it more suited for picture encryption.

## 3. Improved DNA Coding

The traditional DNA-encoding image encryption algorithm consists of three stages: encoding, base operation, and decoding. The base operation consists mostly of the XOR operation, addition operation, and subtraction operation. However, because of the fixed base complementary pairing requirement and base operation criterion of DNA coding, the anti-brute-force attack capacity of the DNA coding algorithm is weak, making security vulnerabilities easy to introduce. In this study, a novel image encryption technique based on the DNA coding strategy is proposed. This technique uses a system function to form the lookup table, decomposes pixel values into row coordinates and column coordinates, and indexes the lookup table to obtain the base pairs stored in the lookup table for base substitution. Several coding methods are merged and interleaved during the substitution process, increasing the randomness and improving the security performance of the algorithm.

### 3.1. DNA Coding

DNA is made up of four deoxynucleotides: adenine (A), cytosine (C), guanine (G), and thymine (T). The chemical structure of the base defines the principle of base complementary pairing, which is two hydrogen bonds between A and T and three hydrogen bonds between G and C. Base operation and permutation can store binary information if a base is represented by a 2-bit binary number. A grayscale image pixel can be represented as an 8-bit binary value, which can be translated into four bases for calculation.

There are a total of 4!=24 base combinations for a pixel based on the complementary pairing of base pairs A↔T and C↔G, and assuming the complementary pairing principle of number pairs is 00↔11 and 01↔10, there are only eight combinations that meet the complementary pairing principle of base pairs, as shown in Table 1:

An encoding mode is selected according to the chaotic sequence value and encodes the pixel value. For example, the pixel value 27 has the binary form of “00011011”. With the first encoding way, four bases “ACGT” can be obtained; if the eighth encoding mode is selected, the encoding is “TGCA”.

### 3.2. Base Substitution

**Figure 2:**
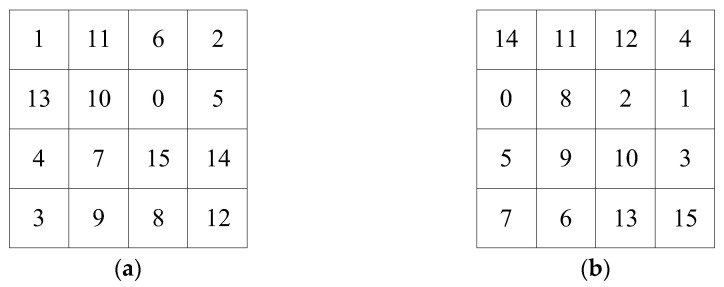
Random matrix: (**a**) matrix 1; (**b**) matrix 2.

The lookup table is generated in the following way: a pseudo-random permutation of integers from 1 to *n* can be generated using the MATLAB function randperm(*n*), whose pseudo-random result is determined by the seed of the random number generator, while the seed of the random number generator can be specified by the function rng(*seed*). When the chaotic value is substituted into the function rng(*seed*) as a seed, the corresponding pseudo-random sequence can be generated. For example, the chaotic values 125 and 201 are taken as the seeds of the random number generator, respectively, to obtain a random number sequence of length 16. Subtract 1 from the sequence value and turn it into matrix 1 and matrix 2, as shown in Figure 2.

**Figure 3:**
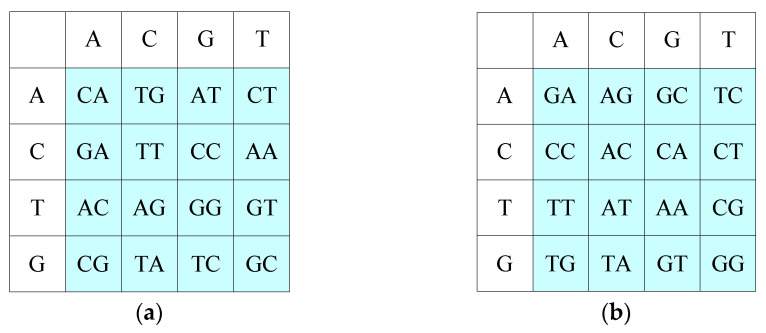
Lookup tables: (**a**) lookup Table 1; (**b**) lookup Table 2.

The two random matrices are encoded by DNA encoding methods based on chaotic values, and lookup Table 1 and lookup Table 2 are obtained. Figure 3 shows the results of matrix 1 and matrix 2 encoded by the third and fifth encoding modes, respectively.

In Figure 3, the blue part is the storage area of the lookup table, in which base pairs are stored, and the colorless part is the row and column coordinates of the lookup table.

The pixel value 27 is encoded to obtain the base “TGCA”; “T” of “TGCA” is taken as the row address of lookup Table 1, and “G” is taken as the column address of lookup Table 1, to obtain the base pair “GC”. “C” is used as the row address of lookup Table 2, and “A” is used as the column address of lookup Table 2 to obtain the base pair “AG”. The search eventually replaces “TGCA” with “GCAG.” Since each pair of bases in the lookup table is unique, the decryption operation can be realized using find(*base*) to obtain the column and row coordinates of the stored values by knowing the stored values in the lookup table.

### 3.3. Base Operation and DNA Decoding

Base operation is based on binary mathematical operation, and its operation rules include XOR, XNOR, addition, and subtraction operations. Take encoding mode 1 as an example: “00” corresponds to base A, and “01” corresponds to base C, if “00” is different from “01” or “01” is the corresponding base C. Table 2 shows the base XOR and XNOR rules in encoding mode 1.

Table 3 shows the operation rules of base addition and subtraction in coding method 1.

The chaotic values in the range 0–255 are encoded to obtain four bases, and four new bases can be obtained by performing base operations with the bases replaced in the previous section.

DNA decoding is the reverse process of DNA coding, which translates the transformed bases into binary numbers in any encoding way to realize the transformation of the pixel values. For example, the pixel value 27 is encoded into “TGCA” in the first encoding mode, and the result obtained through substitution and base operation is “GCAG”. The result obtained through reverse decoding in the second encoding mode is “01100001”, which is the decimal number 97.

## 4. Three-Dimensional and Six-Way Diffusion Strategy

The above-mentioned DNA coding approach can only change pixel values point-by-point, and cannot transmit the influence of a single pixel to the entire world. The traditional diffusion method combines forward diffusion and reverse diffusion, converting the original image into a one-dimensional sequence before performing multiple rounds of replacement, based on the encrypted image grouping link mode, to complete the global diffusion of pixel information on the encrypted image. Study [28] developed a two-dimensional and four-way rapid diffusion approach that used row and column vectors as computation units to boost diffusion efficiency. The approach was improved in this study to make the four-way diffusion algorithm of a single plane applicable to the diffusion between the three channels of a color image, and realizes the avalanche effect that the change in any pixel value can cause the change in the pixel value of the three planes.

### 4.1. Two-Dimensional and Four-Way Diffusion

**Figure 4:**
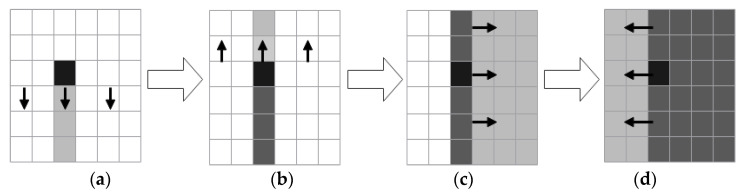
Matrix two-dimensional and four-way diffusion diagram: (**a**) downward diffusion; (**b**) upward diffusion; (**c**) rightward diffusion; (**d**) leftward diffusion.

According to the algorithm provided in study [28], four sequences of length M, M, N, and N are intercepted from the chaotic sequence and employed as the initial diffusion sequences in the right, left, upper, and lower diffusion directions, respectively. At the same time, the chaotic sequence of length M×N is changed into a chaotic matrix of size M×N, which serves as the foundation for four-way diffusion. According to study [28], row vector and column vector were used as the units of measurement, in conjunction with the beginning sequence and the basis, and four repetitions of diffusion were carried out sequentially through the front, back, left, and right. Figure 4 depicts the algorithm flow.

As shown in Figure 4, the value of every pixel in a grayscale image can be diffused across the entire matrix, following two-dimensional and four-way diffusion.

### 4.2. Three-Dimensional and Six-Way Diffusion

**Figure 5:**
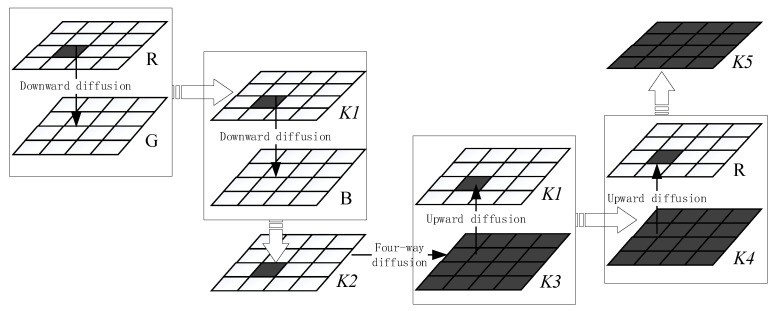
Three-dimensional and six-way diffusion diagram.

The combination of diffusion between planes with two-dimensional and four-way diffusion results in three-dimensional and six-way diffusion. Using the pixel points at the appropriate places of the three channels R, G, and B as an example, the chaotic sequence *x*, *y*, *z*, and *w* with length M×N is also turned into a chaotic matrix of size M×N, as shown in Figure 5.

With chaotic matrix *x* as the basis and color image R and G channels as the unit of operation, the diffusion matrix *K*1 is obtained by top-down diffusion through Equation (4):

(4) $$\{\begin{array}{c} tmp1=\mathrm{mod}\left(x+R,256\right)\\ tmp2=\mathrm{mod}\left(x+G,256\right)\\ K1=\mathrm{bitxor}\left(tmp1,tmp2\right) \end{array},$$

With chaotic matrix *y* as the basis and channels *K*1 and B as the units of operation, the diffusion matrix *K*2 is obtained by top-down diffusion through Equation (5):

(5) $$\{\begin{array}{c} tmp1=\mathrm{mod}\left(y+K1,256\right)\\ tmp2=\mathrm{mod}\left(y+B,256\right)\\ K2=\mathrm{bitxor}\left(tmp1,tmp2\right) \end{array},$$

According to the method of [28], the matrix *K*2 is diffused in two dimensions and four directions to obtain the matrix *K*3.

Using the chaotic matrix *z* as the base and the two matrices *K*3 and *K*1 as the operation units, bottom-up diffusion is performed through Equation (6) to obtain the diffusion matrix *K*4.

(6) $$\{\begin{array}{c} tmp1=\mathrm{mod}\left(z+K1,256\right)\\ tmp2=\mathrm{mod}\left(z+K3,256\right)\\ K4=\mathrm{bitxor}\left(tmp1,tmp2\right) \end{array},$$

Taking the chaotic matrix *w* as the base, and the two matrices *K*4 and R as the units of measurement, bottom-up diffusion is carried out through Equation (7) to obtain the diffusion matrix *K*5.

(7) $$\{\begin{array}{c} tmp1=\mathrm{mod}\left(w+K4,256\right)\\ tmp2=\mathrm{mod}\left(w+R,256\right)\\ K5=\mathrm{bitxor}\left(tmp1,tmp2\right) \end{array},$$

The matrices *K*5, *K*4, and *K*3 are used to replace the original R, G, and B three channels to synthesize a color image. Thus far, the color image’s three-dimensional and six-way diffusion is complete, and changes to pixels in any channel of the color image can affect pixels in all channels.

Equation (8) shows the inverse diffusion of the upper and lower diffusions of the channel during the decryption process:

(8) $$\{\begin{array}{c} tmp1=\mathrm{mod}\left(X+X1,256\right)\\ tmp2=\mathrm{bitxor}\left(tmp1,X2\right)\\ Y=\mathrm{mod}\left(tmp2-X,256\right) \end{array},$$

where *X* is the basis of the current diffusion matrix. Firstly, the three channels *K*5, *K4,* and *K*3 are extracted from the encrypted image, and matrices *K*5 and *K*4 can be substituted into formulas *X*1 and *X*2 to solve the matrix R, where X=w. Substituting *K*4 and *K*3 into *X*1 and *X*2 solves the matrix *K*1, where X=z. The matrix *K*3 is reduced to matrix *K*2 via inverse four-way diffusion. Substituting *K*2 and *K*1 into *X*1 and *X*2 solves the matrix B, where X=y. Then, matrices *K*1 and R are substituted into *X*1 and *X*2 to obtain matrix G, where X=x. The reductions in matrices R, G, and B are realized.

Reference [28] pointed out that the time complexity of the algorithm in the gray image diffusion process is only *O*(2 M + 2 N), which has obvious advantages compared with the complexity of the existing algorithm *O*(2 MN). In this research, the algorithm was extended to three-dimensional space so that color images are also applicable, and the corresponding time complexity is only *O*(2 M + 2 N + 4), while the time complexity of study [28] was *O*(3 × (2 M +2 N)). If the grayscale image is divided into blocks and then diffused using the three-dimensional six-direction algorithm, the algorithm’s time complexity is smaller.

## 5. Encryption Procedure

### 5.1. Image Encryption and Preprocessing

Before picture encryption, the average pixel values of the three channels, R, G, and B, were determined and quantized into a fractional *mean* in the range of 0–1. Its calculating formula is shown in Equation (9):

(9) $$mean=\frac{\sum_{i=0}^{M-1}\sum_{j=0}^{N-1}p_{ij}}{M\times N\times 256},$$

where M and N are the plaintext image’s length and width, *i* and *j* are pixel coordinates, and *p* are pixel points in the equation.

The mean value of each channel is taken as the initial values *x*0, *y*0, and *z*0 of the four-dimensional hyperchaotic system. The value of *w*0 is set by the user. The chaotic system is pre-iterated 800 times to eliminate the transient effect and make the system enter the chaotic state entirely. Then, it iterates 2 × M × N times to produce four chaotic sequences *x*, *y*, *z*, and *w* of length 2 × M × N. The first 2 × M × N sequence values are intercepted to obtain *x*1, *y*1, *z*1, and *w*1, which are used to realize DNA coding. The last M × N sequence values *x*2, *y*2, *z*2, and *w*2 are used for three-dimensional six-way diffusion.

Equation (10) is used to modulo the chaotic sequences *x*1, *y*1, *z*1, and *w*1 against 2^24^ to obtain the sequence *X*1, *Y*1, *Z*1, and *W*1.

(10) $$X=\mathrm{mod}\left(floor\left(abs\left(x\times 10^{15}\right)\right),2^{24}\right),$$

The chaotic sequences *x*2, *y*2, *z*2, and *w*2 are quantified by Equation (11) to obtain the sequences *X*2, *Y*2, *Z*2, and *W*2, which are used to realize three-dimensional and six-way diffusion.

(11) $$X=\mathrm{mod}\left(floor\left(abs\left(x\times 10^{13}\right)\right),256\right),$$

### 5.2. Image Encryption Process

**Figure 6:**
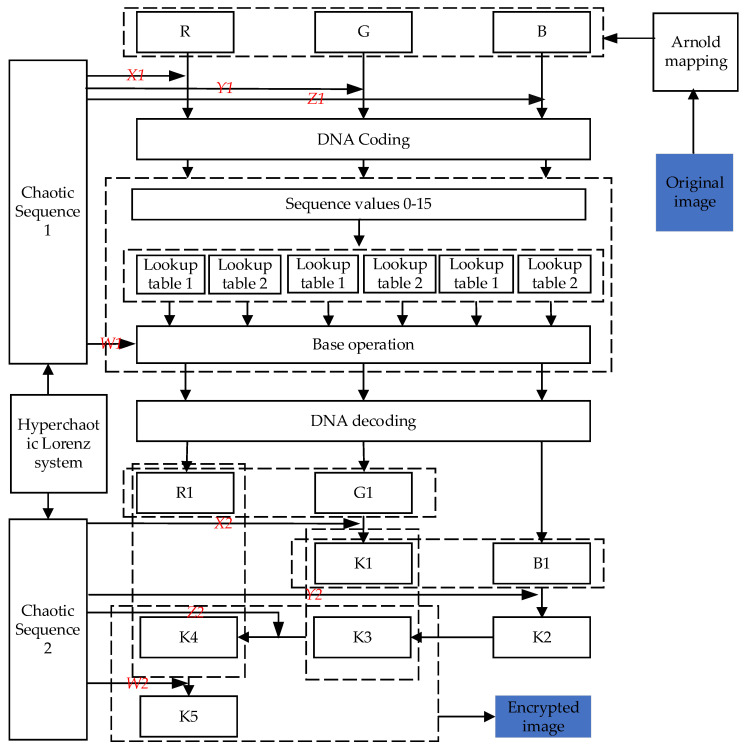
Encryption flow chart.

The encryption algorithm procedure described in this study is separated into three stages: scrambling, DNA coding, and three-dimensional and six-way diffusion. Figure 6 depicts the specific flow chart.

In order to better describe the encryption flow chart, the following is divided into eight steps for a specific description.

**Step 1:** Obtain the initial values of the four-dimensional hyperchaotic system *x*0, *y*0, and *z*0 from the original image, and set w0=4.4, system parameters a=10, b=8/3, c=28, and r=−1. According to the preprocessing process mentioned in Section 5.1, the chaotic sequences *X*1, *Y*1, *Z*1, and *W*1, and *X*2, *Y*2, *Z*2, and *W*2 are obtained.

**Step 2:** Set Arnold mapping coefficients a=2, b=2, and the number of scrambles n=110, and then scramble channels R, G, and B of the original image, respectively.

**Step 3:** Generate lookup Table 1 and lookup Table 2. Convert *X*1 to a 24-bit binary number. Take *X*1[24:20] as the seed of the random number generator, then generate matrix 1 using the function randperm(*seed*), and encode matrix 1 by the encoding mode selected by *X*1[19:17] to obtain lookup Table 1; *X*1[16:12] is used as the seed of the random number generator, and generates matrix 2 via the function randperm(*seed*), and the encoding mode chosen by *X*1[11:9] encodes matrix 2 to obtain lookup Table 2. Similarly, sequences *Y*1 and *Z*1 are used for the lookup table generation of G and B channels.

**Step 4:** *X*1[8:6] selects the encoding method to encode the pixel value of the R channel and obtains four bases. The first two bases are used as the row coordinates and column coordinates of lookup Table 1, and replace index values with base pairs stored in a lookup table. The last two bases act on lookup Table 2 for indexing and replacing. Similarly, the sequences *Y*1 and *Z*1 act on the G channel and B channel to achieve base substitution.

**Step 5:** Convert *W*1 to a 24-bit binary number. Cut the sequence *W*1 into *W*1[24:17], *W*1[16:9], and *W*1[8:1]. *X*1[5:4] selects the encoding method to encode *W*1[24:17], and the encoded base selects the base operation method according to *X*1[2:1] and performs base operation with the replacement value in the previous step. *W*1[16:9] and *W*1[8:1] do the same with chaos *Y*1 and *Z1.*

**Step 6:** According to *X*1[3:1], the encoding method is selected, and the base after the base operation is decoded into binary and converted to decimal to obtain the pixel value R1. G1 and B1 are obtained in the same way according to the sequence values *Y*1 and *Z*1.

**Step 7:** According to the three-dimensional and six-way diffusion strategy proposed in this study, chaotic sequences *X*2, R1, and G1 are used to obtain matrix *K*1, and chaotic sequences *Y*2, *K*1, and B1 are used to obtain matrix *K*2. *K*2 is diffused in two dimensions to obtain *K*3. The chaotic sequences *Z*2, *K*3, and *K*1 are used to obtain matrix *K*4, and the chaotic sequences *W*2, *K*4, and R1 are used to obtain matrix *K*5.

**Step 8:** Synthesize the *K*3, *K*4, and *K*5 channels into a color ciphertext image.

## 6. Simulation Experiment and Performance Analysis

This study used color images with sizes 512 × 512 as the original images for testing the algorithm performance, which mainly included images Lena, Peppers, and Baboon. The algorithm’s key space, key sensitivity, differential attack resistance, histogram, adjacent pixel correlation, information entropy, and robustness were evaluated and compared.

### 6.1. Experimental Simulation Results

**Figure 7:**
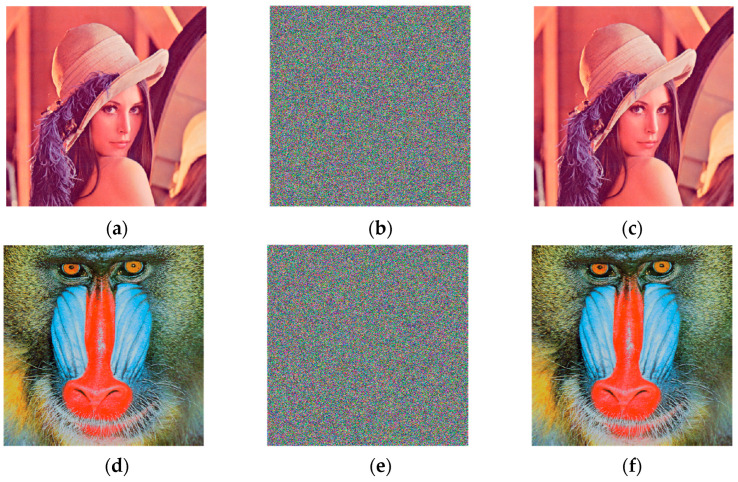

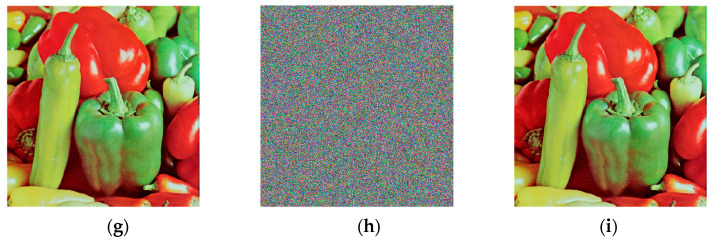
Encryption and decoding results: (**a**) Lena original image; (**b**) Lena encryption image; (**c**) Lena decryption image; (**d**) Baboon original image; (**e**) Baboon encryption image; (**f**) Baboon decryption image; (**g**) Peppers image; (**h**) Peppers encryption image; (**i**) Peppers decryption image.

Figure 7 depicts the results of the algorithm’s encryption and decryption of Lena, Peppers, and Baboon color images.

As seen in Figure 7, the encrypted images in Figure 7b,e,h are jumbled, and it is impossible to see any information from the original image with the naked eye. The decoded photos in Figure 7c,f,i were compared to the corresponding original images without missing data, allowing the original images to be recovered without loss.

### 6.2. Sensitivity Analysis

#### 6.2.1. Key Space Analysis

The key space refers to the set of all possible keys that can be used to generate the key. The size of the key space depends on the length of the security key, and is one of the most important characteristics that determine the strength of the cryptosystem. The key space required by the encryption algorithm to effectively resist brute force attacks is at least 2^100^. The algorithm key proposed in this paper includes Arnold scrambling coefficients a=2, b=2, and scrambling number n=110; the initial values *x*0, *y*0, *z*0 of the four-dimensional hyperchaotic system are calculated by the original image, and the initial value w0=4.4 is set by oneself. Moreover, the system coefficient numbers are a=10, b=8/3, c=28, and r=−1. If the accuracy of the computer is $10^{-15}$, the key space of the encryption algorithm proposed in this paper is at least ${\left(10^{15}\right)}^{8}=10^{120}$ and much larger than 2^100^; thus, the algorithm can effectively prevent brute force attacks.

#### 6.2.2. Key Sensitivity Analysis

As a result of key sensitivity, a tiny change in the key can result in an entirely different encryption result. The key sensitivity of chaotic cryptography includes the sensitivity of the initial state of the chaotic system and the sensitivity of the control parameters. Sensitivity is assessed using two parameters: pixel number rate of change (NPCR) and uniform average change intensity (UACI). Assuming that *C*1 is the encrypted picture corresponding to the original image and *C*2 is the encrypted image after the key is changed, NPCR and UACI denote the number of changing pixels and the average number of changing intensities between two encrypted images, *C*1 and *C*2. Their corresponding ideal values are NPCR=99.6094% and UACI=33.4635%, respectively. The calculation equation is as follows:

(12) $$\mathrm{NPCR}=\frac{\sum_{i,j}D\left(i,j\right)}{M\times N}\times 100\%,$$

where M and N are the width and height of the image, and *i* and *j* are the index values of the rows and columns. The variable D(i, j) is defined as Equation (13):

(13) $$D\left(i,j\right)=\{\begin{array}{c} 1,C1\left(i,j\right)\neq C2\left(i,j\right)\\ 0,C1\left(i,j\right)=C2\left(i,j\right) \end{array},$$

Accordingly, UACI can be used to measure the mean value of the contrast intensity of the color component, and its formula is shown in Equation (14):

(14) $$\mathrm{UACI}=\frac{1}{M\times N}\frac{\sum \left(C1\left(i,j\right)-C2\left(i,j\right)\right)}{255},$$

**Figure 8:**
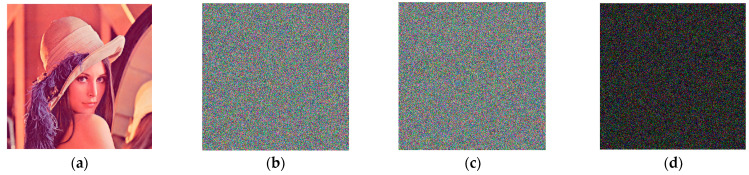
Sensitivity of initial vector *x*0: (**a**) Lena; (**b**) encrypted image *C*1 at *x*0; (**c**) encrypted image *C*2 at $x0=x0+10^{-14}$; (**d**) difference between (**b**,**c**).

**Figure 9:**
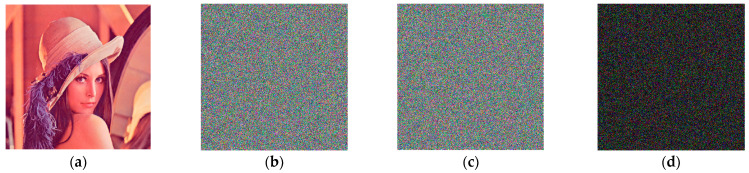
Sensitivity of initial vector *w*0: (**a**) Lena; (**b**) encrypted image *C*1 at w0=4.4; (**c**) encrypted image *C*2 at $w0=4.4+10^{-14}$; (**d**) difference between (**b**,**c**).

**Figure 10:**
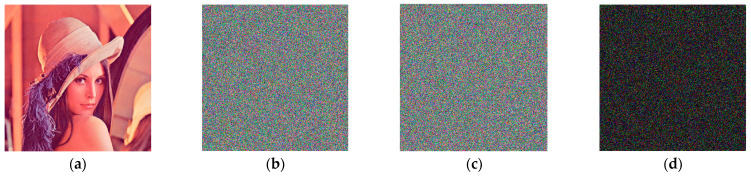
Sensitivity of initial vector *a*: (**a**) Lena; (**b**) encrypted image *C*1 at a=2; (**c**) encrypted image *C*2 at a=3; (**d**) difference between (**b**,**c**).

From the encryption level, Figure 8, Figure 9 and Figure 10 show the encrypted image *C*1 of Lena, the encrypted image *C*2 after the disturbance of key *x*0 and *w*0 is increased by 10^−14^, and the disturbance of scrambling coefficient *a* is increased by 1, as well as the difference between *C*1 and *C*2 of the encrypted image.

From the difference image (d), it can be seen that the encrypted image *C*2 is completely different from the encrypted image *C*1 after the disturbance of key *x*0 and *w*0 is increased by 10^−14^, and the disturbance of scrambling coefficient *a* is increased by 1.

Table 4 shows the NPCR and UACI values between *C*1 and *C*2 of encrypted images after the perturbation of different keys is added, respectively.

**Figure 11:**
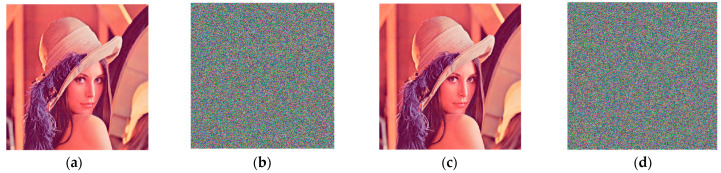
Decrypted image after key perturbation: (**a**) Lena; (**b**) encrypted image when w0=4.4; (**c**) decrypted image when w0=4.4; (**d**) decrypted image when $w0=4.4+10^{-14}$.

From the data provided in Table 4, it can be observed that when the initial values of the chaotic system *x*0, *w*0 increase by 10^−14^ disturbance and the Arnold scrambling coefficient *a* increases by 1, the NPCR and UACI values between the corresponding ciphertext image and the original ciphertext image are close to ideal values. The minimum difference between the NPCR value of each channel and the ideal value of 99.6094% is 0.0001%, and the maximum difference is 0.0138%. The maximum difference between UACI and the ideal value of 33.4635% is 0.1045%, and the minimum difference is 0.0151%. It shows that the corresponding ciphertext image after the minimum precision perturbation of the key of the algorithm changes greatly compared with the original ciphertext image, which proves that the key sensitivity of the algorithm is very strong. From the perspective of the decryption level, when encrypting, the key provided by the algorithm is used to encrypt, and when decrypting, the key *w*0 is increased by 10^−14^ perturbation and then the decryption operation is performed. The results are shown in Figure 11.

Figure 11c,d show that when the difference between the key and the original key during decryption is 10^−14^, the decrypted image is absolutely irrelevant to the encrypted image. As a result of the decryption level analysis, the key to this algorithm is quite sensitive.

### 6.3. Differential Attack

The differential attack is a powerful approach for cracking encrypted images. It means that the attacker makes subtle changes to the original image data, encrypts it with the proposed encryption algorithm, determines the relationship between the original image data and the encrypted image data by comparing the two encrypted images, and uses this relationship and rule to crack the encrypted image. A differential attack is a type of ciphertext attack. The performance of the anti-differential attack is determined by the initial image sensitivity. NPCR and UACI can also be used to calculate the size difference between two encrypted images. When the difference is bigger, the NPCR and UACI are closer to the ideal values of 99.6094% and 33.4635%, respectively, indicating that the anti-differential attack capability of the system is stronger.

In order to test the anti-differential attack performance of the algorithm, the Lena color image with a size of 512 × 512 was selected for detection and comparison. First, 100 pixels were randomly selected in any channel of the image, and a pixel value was added or subtracted in turn to fine-tune; then, the fine-tuned plaintext image was encrypted using the algorithm proposed in this study to obtain the ciphertext *C*2; then, the original ciphertext substituted text *C*1 and fine-tuned ciphertext *C*2 into Equations (13)–(15), the NPCR and UACI values of the two were calculated each time, and finally the average values of NPCR and UACI were calculated 100 times. The comparison of test results with other algorithm results is shown in Table 5.

In order to reflect the anti-differential attack ability of the algorithm in this research, Table 5 provides the NPCR and UACI values of different algorithms. By observing the data, it can be found that each algorithm’s NPCR and UACI values are close to the ideal values of 99.6094% and 33.4635%, indicating that each algorithm has a better ability to resist differential attacks. In order to further compare the performance of the different algorithms, the average difference between the NPCR and UACI of the R, G, and B channels and the ideal value was calculated. It can be found that the average difference between the NPCR and the ideal value of the algorithm in this study is 0.00036, and the average difference between UACI and the ideal value is 0.0211; study [29] corresponds to 0.0139 and 0.0131; study [30] corresponds to 0.0010 and 0.0464; study [31] corresponds to 0.0089 and 2.9959; study [31] corresponds to 0.0115 and 3.1368. From this, it can be found that this algorithm’s NPCR and UACI values are closer to the ideal values than those of the other encryption algorithms. This shows that after fine-tuning a specific pixel of the plaintext image, the algorithm achieves a more significant transformation of the ciphertext image, which proves that the algorithm is more resistant to differential attacks.

### 6.4. Statistical Analysis

#### 6.4.1. Histogram Analysis

**Figure 12:**
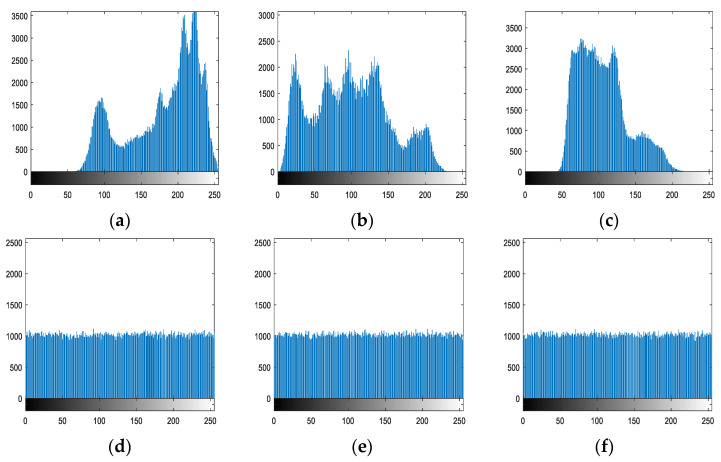
Histogram of color original image and encrypted image of Lena: (**a**) R channel of the original image; (**b**) G channel of the original image; (**c**) B channel of the original image; (**d**) R channel of the encrypted image; (**e**) G channel of the encrypted image; (**f**) B channel of the encrypted image.

The histogram displays the image’s statistical data, which can intuitively indicate the distribution of each gray value in the image. The histograms of the original image show clear statistical trends. The statistical analysis attacker can compare the ciphered image to its statistical law and determine the transformation relationship between the original and ciphered images. To withstand statistical attacks, the encrypted image’s histogram must be uniform and completely different from the original image’s histogram. Figure 12 depicts the histograms of the three channels R, G, and B of the original and encrypted Lena images.

Figure 12 shows that the three channels of the encrypted image, R, G, and B, are all near a horizontal line, and are completely distinct from the original image, which may effectively resist statistical attacks.

#### 6.4.2. Correlation Analysis

Correlation analysis involves the examination of two or more correlated variable elements in order to determine the degree of similarity between variables. Since the association between neighboring pixels in original photographs is very strong, leaking a pixel will result in the leakage of surrounding pixel information. This feature can be used by attackers to infer the pixel value surrounding the leaked pixel. To resist statistical attacks, a decent encryption method can disrupt the correlation of each pixel in the original image. Correlation coefficients in the horizontal, vertical, and diagonal directions are included in the correlation measure. In general, the correlation of the original image’s neighboring pixels is close to one, whereas the correlation of the original image’s neighboring pixels is close to zero. Equation (15) shows its formula:

(15) $$\{\begin{array}{c} R_{\mathrm{xy}}=\frac{\mathrm{cov}\left(x,y\right)}{\sqrt{D\left(x\right)}\sqrt{D\left(y\right)}}\\ E\left(x\right)=\frac{1}{L}\sum_{i=1}^{L}\left(x_{i}\right)\\ D\left(x\right)=\frac{1}{L}\sum_{i=1}^{L}{\left(x_{i}-E\left(x\right)\right)}^{2}\\ \mathrm{cov}\left(x,y\right)=\frac{1}{L}\sum_{i=1}^{L}\left(x_{i}-E\left(x\right)\right)\left(y_{i}-E\left(y\right)\right) \end{array},$$

where *x* and *y* are the two adjacent pixels, *L* is the total number of pixels in the image, $R_{xy}$ is the correlation between two adjacent pixels, cov(x,y) is the covariance of two pixels, $\sqrt{D\left(x\right)}$ is the standard deviation, and E(x) is the mean value.

**Figure 13:**
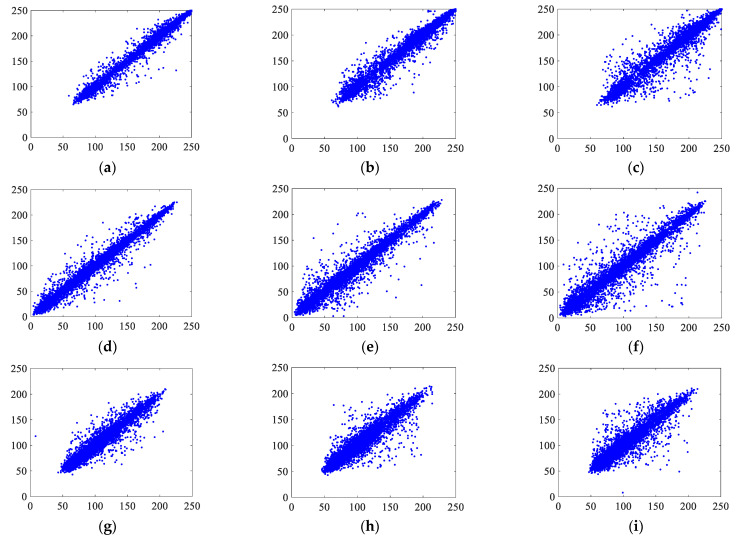
Pixel pair distributions of Lena: (**a**–**c**) are the distributions of pixel pairs in the horizontal, vertical, and diagonal directions of channel R of the original image, respectively; (**d**–**f**) are the distributions of pixel pairs in the horizontal, vertical, and diagonal directions of channel G of the original image, respectively; (**g**–**i**) are the distributions of pixel pairs in the horizontal, vertical, and diagonal directions of channel B of the original image, respectively.

**Figure 14:**
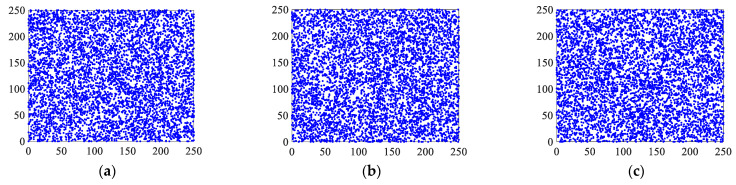

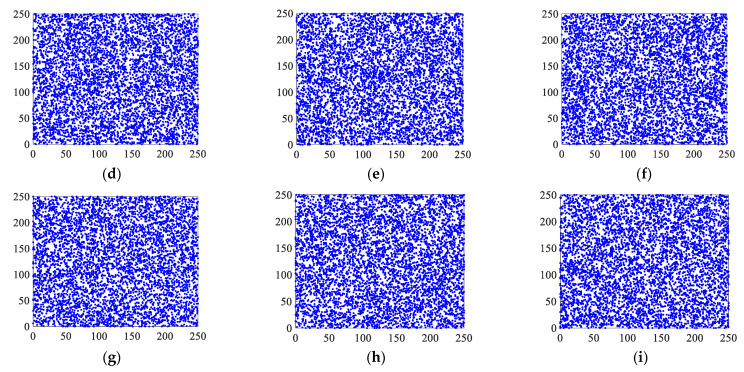
Pixel pair distributions of encrypted image: (**a**–**c**) are the distributions of pixel pairs in horizontal, vertical, and diagonal directions of channel R of the encrypted image, respectively; (**d**–**f**) are the distributions of pixel pairs in horizontal, vertical, and diagonal directions of channel G of the encrypted image, respectively; (**g**–**i**) are the distributions of pixel pairs in the horizontal, vertical, and diagonal directions of the encrypted image G channel, respectively.

Randomly, 5000 pixels in the plaintext and ciphertext images were selected, and the distributions of these pixels in the horizontal, vertical, and diagonal directions from the R, G, and B channels were observed. Figure 13 and Figure 14 show the plaintext and ciphertext distributions of the pixel points, respectively.

The correlations between the original image and the encrypted image in Figure 13 and Figure 14 show that among the 5000 randomly selected pixel pairs, the pixel pairs in each direction of the three channels of the original image are closely distributed near a diagonal line, whereas the pixel pairs of the encrypted image are scattered and essentially irrelevant. The horizontal, vertical, and diagonal correlation coefficients of three sets of color encrypted images are shown in Table 6, along with a comparison to other techniques.

It can be seen from the data that the correlations of the pixel pairs of the ciphertext image corresponding to the algorithm in this study are less than 0.01 in the horizontal direction, vertical direction, and diagonal direction. This shows that the correlation between each pixel of the ciphertext is very low, which can effectively resist statistical attacks. Compared with the other algorithms, it can be found that the correlations of the pixel pairs of the Lena ciphertext image corresponding to the algorithm in this study are generally smaller than those of other literature, indicating that the statistical characteristics of the ciphertext image are lower, which proves that the algorithm has a stronger ability to resist statistical attacks.

#### 6.4.3. Information Entropy Analysis

Entropy is generally used to describe the complexity of things, and entropy is a measure of the random degree of information. The ideal value of entropy for a color image with a pixel value domain of [0, 255] is 8. The closer the entropy value is to 8, the higher the average uncertainty and complexity of the signal, and the better the encryption algorithm. The calculation formula of information entropy is shown in Equation (16):

(16) $$H\left(x\right)=-\sum_{i=1}^{L}P\left(x_{i}\right)\log_{2}P\left(x_{i}\right),$$

where, $x_{i}$ is the gray value, and $p\left(x_{i}\right)$ is the probability of gray level $x_{i}$ appearing.

Table 7 lists the entropy values of the three channels of the Lena, Peppers, and Baboon original images and encrypted images, respectively, and uses the Lena color image to compare with other algorithms.

From Table 7, we can see that the entropy values of the three channels of the ciphered image are above 7.9992, which is close to the ideal value of 8. This indicates that ciphertext image encrypted by this algorithm has high complexity and high uncertainty.

### 6.5. Robustness Analysis

The encrypted image will invariably be damaged by noise pollution or information loss during Internet transmission, making it difficult to decipher the decrypted image. As a result, an encryption algorithm must be resilient and able to withstand noise pollution and the loss of some information in real life. To demonstrate the robustness of the algorithm, noise attack and cropping attack were carried out on the Lena color ciphertext image before decryption.

#### 6.5.1. Salt-Pepper Noise Attack

**Figure 15:**
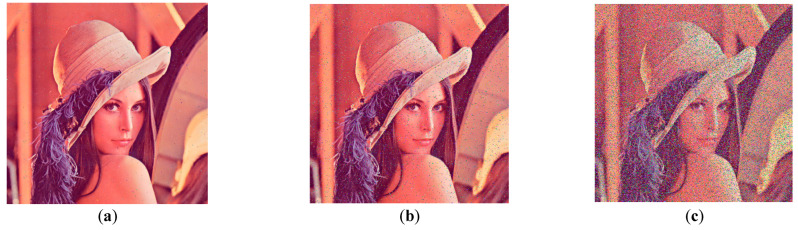
Noise attack: (**a**) decrypted image with density of 0.001; (**b**) decrypted image with density of 0.01; (**c**) decrypted image with density of 0.1.

Salt and pepper noise was added to the Lena encryption image, with densities of 0.001, 0.01, and 0.1. Figure 15 depicts the decryption impact of the encrypted image after noise addition.

Figure 15 shows that the quality of the decrypted image degrades as the density of the salt and pepper noise increases. When the density of the salt and pepper noise is 0.001, there are only a few dispersed noise points in the decrypted image. However, when the density is 0.1, there are many noise points in the decrypted image, affecting the image’s information reading.

The most popular and extensively used objective metric of image quality is PSNR, with higher values signifying greater image quality. When the PSNR value is greater than 40 dB, the image quality is exceptional; when it is 30–40 dB, the image quality is good; when it is 20–30 dB, the image quality is bad but acceptable; and when it is less than 20 dB, the image quality is undesirable. The following is how PSNR is defined:

(17) $$\{\begin{array}{c} \mathrm{MSE}=\frac{1}{M\times N}\sum_{i=1}^{M}\sum_{j=1}^{N}{\left(P\left(i,j\right)-D(i,j)\right)}^{2}\\ \mathrm{PSNR}=10\log_{10}\left(\frac{{\left(2^{n}-1\right)}^{2}}{\mathrm{MSE}}\right) \end{array},$$

where MSE represents the mean square error of the current image *p* and the reference image *D*, M and N are the height and width of the image, respectively, and *n* is the number of pixels in bits.

Equation (17) was used to test the decryption diagram of salt and pepper noise added to different degrees, and the test results are shown in Table 8:

Table 8 shows that when the salt and pepper noise level is 0.00001, the PSNR of the three channels is better than 50 dB, indicating that the decrypted image quality is excellent at this moment. When the salt and pepper noise density is 0.0001, and the PSNR of the three channels is larger than 40 dB, the decrypted image quality is good at this moment. The PSNR of the three channels is greater than 30 dB when the salt and pepper noise density is 0.001, indicating that the decrypted image quality is average at this time. The PSNR of the three channels is larger than 20 dB when the salt and pepper noise density is 0.01, indicating that the decrypted image quality is bad at this time. The anti-noise ability of the algorithm is acceptable when the salt and pepper noise density is less than 0.01.

#### 6.5.2. Gaussian Noise Attack

Gaussian noise with a mean of 0 and variances of 0.001, 0.005, and 0.01 was added to the Lena encrypted image. Table 9 shows the PSNR values of each channel after decryption.

**Figure 16:**
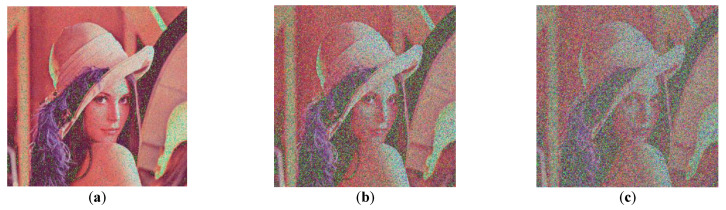
Gaussian noise attack: (**a**) decrypted image with variance of 0.001; (**b**) decrypted image with variance of 0.005; (**c**) decrypted image with variance of 0.01.

Figure 16 shows the decrypted image. The results show that the decrypted image can still be recognized under a certain degree of noise attack.

#### 6.5.3. Tailoring Attacks

In order to show the decryption ability of the algorithm for flossy images, this study trimmed the upper left corner and the center of the encrypted image, and the sizes were 32×32, 64×64, 128×128, and 256×256, in order.

**Figure 17:**
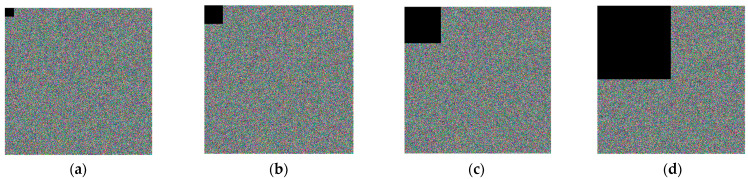

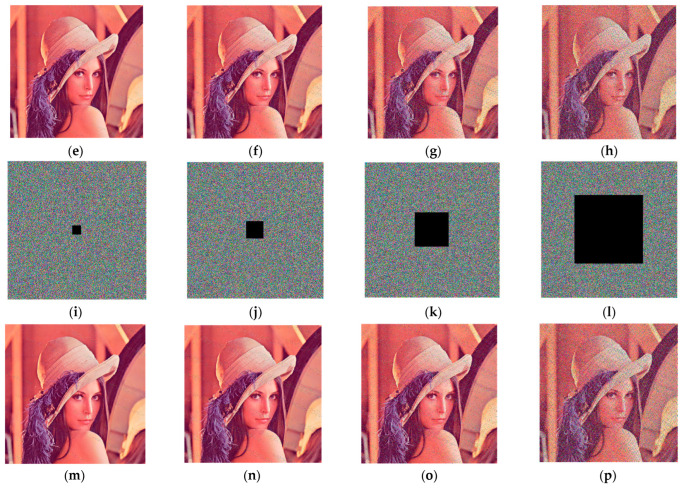
Clipping attack diagram. (**a**–**d**) clipping the upper left corner of the encrypted image; (**e**–**h**) is the decrypted image after clipping the upper left corner; (**i**–**l**) trims the central position of the encrypted image; and (**m**–**p**) is the encrypted image after the central position trims.

As illustrated in Figure 17, the varied crop placements and crop sizes had an effect on the degree of decryption restoration. The worse the image reproduction, the higher the crop size. Even though the ciphered image loses a quarter of its image information, the broad outline of the original image may be reconstructed, indicating that the approach is resistant to cropping attacks.

### 6.6. Efficiency Analysis

In addition to security considerations, algorithm efficiency is also an important aspect of a good encryption algorithm. Time complexity is a measure of efficiency. Compared with classical DNA coding, the improved DNA coding proposed in this study uses an alternative process, and the increased time complexity mainly includes the generation of lookup tables and indexes on lookup tables. Assuming that the image size is m×n, the time complexity of generating the lookup table is *O*(4 *mn*), and the time complexity required for indexes is *O*(*mn*). In the diffusion stage, the classical diffusion algorithm needs to transform the image into a one-dimensional sequence for forward and reverse diffusion, so the time complexity of the color image is *O*(6 *mn*). In this research, a three-dimensional and six-way algorithm was adopted. Under the premise of parallel operation, only *O*(2 *m* + 2 *n* + 4) times are needed, which improves the diffusion efficiency.

## 7. Conclusions

Through research on image encryption algorithms, it was found that there are some defects in the DNA code encryption algorithm and the traditional one-dimensional diffusion image encryption algorithm. To address the problem that the DNA coding algorithm is weak in resisting exhaustive attacks and prone to safety hazards due to its fixed base complementary pairing criteria and base operation criteria, this study proposed an improved DNA coding method, using a lookup table to perform base substitutions. In this method, the chaotic sequence was used as the seed of the pseudo-random sequence generator to regenerate the random sequence and encode it into a lookup table. At the same time, the plaintext pixel values were encoded as the row and column coordinates of the lookup table, and the base pairs stored in the lookup table were obtained through the index to replace the plaintext pixel values. This base replacement method, which is based on the chaotic system to generate pseudo-random sequences twice and form a lookup table, improves the complexity and randomness of the algorithm through the interspersed use of various encoding methods, thereby enhancing the security performance of the algorithm. To address the problem that the traditional one-dimensional diffusion algorithm is not efficient when encrypting images with a large amount of data, a three-dimensional scrambling diffusion algorithm was proposed, which takes the matrix, row, and column as the diffusion unit successively, and improves the diffusion efficiency through the parallel operation. To sum up, it is of great value to study the image encryption algorithm based on DNA encoding; the improved DNA-encoding and three-dimensional six-direction diffusion algorithm of this algorithm can be realized through parallel computing, which can improve security performance and improve operating efficiency at the same time. Especially for the use of hardware such as FPGA to process large color images, this research has great significance.

## Figures and Tables

**Table 1:** DNA coding rules.

	1	2	3	4	5	6	7	8
00	A	A	C	G	C	G	T	T
01	C	G	A	A	T	T	C	G
10	G	C	T	T	A	A	G	C
11	T	T	G	C	G	C	A	A

**Table 2:** Base XOR, XNOR operations.

⊕	A	G	C	T	⊙	A	G	C	T
A	A	C	G	T	A	T	C	G	A
G	C	A	T	G	G	C	T	A	G
C	G	T	A	C	C	G	A	T	C
T	T	G	C	A	T	A	G	C	T

**Table 3:** Base addition and subtraction.

+	A	T	C	G	-	A	T	C	G
G	G	C	T	A	A	A	T	C	G
C	C	A	G	T	G	G	C	T	A
T	T	G	A	C	C	T	G	A	T
A	A	T	C	G	T	C	A	G	C

**Table 4:** NPCR and UACI for changing key values.

Key Handling	Channel	NPCR	UACI
$x0+10^{-14}$	R	99.6040%	33.4196%
	G	99.5949%	33.5279%
	B	99.6041%	33.4355%
$w0+10^{-14}$	R	99.6140%	33.5680%
	G	99.6178%	33.4946%
	B	99.5872%	33.3919%
a+1	R	99.6231%	33.4900%
	G	99.5995%	33.4260%
	B	99.6334%	33.3827%

**Table 5:** Performance indicators against differential attacks.

	NPCR			UACI		
Lena	R	G	B	R	G	B
Algorithm of this study	99.6094	99.6055	99.6122	33.4511	33.4850	33.5177
Study [29]	99.65	99.52	99.70	33.43	33.49	33.51
Study [30]	99.6078	99.6140	99.6033	33.4457	33.5598	33.5243
Study [31]	99.6021	99.6068	99.5926	33.0861	30.6170	27.6997
Study [32]	99.6001	99.5911	99.6025	33.0273	30.3115	27.6413

**Table 6:** Correlation comparison.

Algorithm	Image Channel		Horizontal	Vertical	Diagonal
Our algorithm	Peppers	R	−2.1153 × 10^−4^	0.0019	0.0033
		G	−3.4458 × 10^−5^	−8.7850 × 10^−5^	−0.0037
		B	0.0013	0.0015	−3.2065 × 10^−4^
	Baboon	R	0.0018	0.0019	−8.4925 × 10^−5^
		G	−5.0995 × 10^−4^	7.6649 × 10^−5^	−0.0046
		B	−3.5015 × 10^−4^	−0.0021	0.0018
	Lena	R	−0.0048	0.0031	−0.0029
		G	0.0016	1.5975 × 10^−4^	−2.4794 × 10^−4^
		B	0.0022	−6.4675 × 10^−4^	−0.0039
Study [29]	Lena	R	−0.0131	0.0142	−0.004
		G	−0.0007	−0.0167	−0.0145
		B	0.0036	0.0083	−0.0214
Study [30]	Lena	R	−0.0003	0.0019	−0.0011
		G	−0.0077	−0.0057	0.0100
		B	0.0194	−0.0037	−0.0023
Study [31]	Lena	R	0.0045	0.0130	−0.0097
		G	0.0040	−0.0026	0.0016
		B	0.0098	0.0029	0.0105

**Table 7:** Comparison of information entropy.

Algorithm	Image		R	G	B
Proposed algorithm	Peppers	original image	7.3388	7.4963	7.0583
		ciphered image	7.9993	7.9993	7.9993
	Baboon	original image	6.9293	6.3175	7.2895
		ciphered image	7.9994	7.9992	7.9993
	Lena	original image	7.2531	7.5940	6.9684
		ciphered image	7.9993	7.9994	7.9993
Study [29]	Lena	ciphered image	7.9993	7.9994	7.9993
Study [30]	Lena	ciphered image	7.9975	7.9970	7.9970
Study [31]	Lena	ciphered image	7.9026	7.9022	7.9030
Study [32]	Lena	ciphered image	7.9994	7.9993	7.9994

**Table 8:** NPCR values of the decryption diagram after noise.

	PSNR (dB)		
noise density	R	G	B
0.00001	50.0124	51.1289	52.0839
0.0001	41.4961	42.2700	41.9721
0.001	32.0578	32.3174	32.1268
0.01	21.9751	22.4952	22.1721

**Table 9:** NPCR values of the decryption diagram after noise.

	PSNR (dB)		
variance	R	G	B
0.001	15.9548	16.3771	21.8248
0.005	10.4744	11.6216	14.5379
0.01	10.1382	10.3563	12.0193

## Data Availability

Not applicable.
